# Mutations in the SARS-CoV-2 RNA dependent RNA polymerase confer resistance to remdesivir by distinct mechanisms

**DOI:** 10.1126/scitranslmed.abo0718

**Published:** 2022-04-28

**Authors:** Laura J. Stevens, Andrea J. Pruijssers, Hery W. Lee, Calvin J. Gordon, Egor P. Tchesnokov, Jennifer Gribble, Amelia S. George, Tia M. Hughes, Xiaotao Lu, Jiani Li, Jason K. Perry, Danielle P. Porter, Tomas Cihlar, Timothy P. Sheahan, Ralph S. Baric, Matthias Götte, Mark R. Denison

**Affiliations:** ^1^ Department of Pediatrics, Vanderbilt University Medical Center, Nashville, TN, 37232, USA; ^2^ Vanderbilt Institute for Infection, Immunology, and Inflammation, Nashville, TN, 37232, USA; ^3^ Department of Medical Microbiology and Immunology, University of Alberta, Edmonton, AB, T6G 2T9, CA; ^4^ Department of Pathology, Microbiology, and Immunology, Vanderbilt University Medical Center, Nashville, TN, 37232, USA; ^5^ Gilead Sciences, Inc, Foster City, CA, 94404, USA; ^6^ Department of Epidemiology, University of North Carolina at Chapel Hill, Chapel Hill, NC, 27599, USA

## Abstract

The nucleoside analog remdesivir (RDV) is a Food and Drug Administration (FDA)-approved antiviral for treatment of severe acute respiratory syndrome coronavirus 2 (SARS-CoV-2) infections. Thus, it is critical to understand factors that promote or prevent RDV resistance. We passaged SARS-CoV-2 in the presence of increasing concentrations of GS-441524, the parent nucleoside of RDV. After 13 passages, we isolated three viral lineages with phenotypic resistance as defined by increases in half-maximal effective concentration (EC_50_) from 2.7-to 10.4-fold. Sequence analysis identified non-synonymous mutations in nonstructural protein 12 RNA-dependent RNA polymerase (*nsp12*-RdRp): V166A, N198S, S759A, V792I and C799F/R. Two lineages encoded the S759A substitution at the RdRp Ser_759_-Asp-Asp active motif. In one lineage, the V792I substitution emerged first, then combined with S759A. Introduction of S759A and V792I substitutions at homologous *nsp12* positions in murine hepatitis virus (MHV) demonstrated transferability across betacoronaviruses; introduction of these substitutions resulted in up to 38-fold RDV resistance and a replication defect. Biochemical analysis of SARS-CoV-2 RdRp encoding S759A demonstrated a roughly 10-fold decreased preference for RDV-triphosphate (RDV-TP) as a substrate, whereas *nsp12*-V792I diminished the uridine-triphosphate (UTP) concentration needed to overcome template-dependent inhibition associated with RDV. The in vitro-selected substitutions identified in this study were rare or not detected in the greater than 6 million publicly available *nsp12*-RdRp consensus sequences in the absence of RDV selection. The results define genetic and biochemical pathways to RDV resistance and emphasize the need for additional studies to define the potential for emergence of these or other RDV resistance mutations in clinical settings.

## Teaser

SARS-CoV-2 develops in vitro resistance to remdesivir by distinct, complementary mutations and mechanisms in the viral RNA-dependent RNA polymerase.

## INTRODUCTION

Severe acute respiratory syndrome coronavirus 2 (SARS-CoV-2) infections have caused more than 850,000 deaths in the United States and over 5 million deaths worldwide (*1*, *2*). Remdesivir (RDV, GS-5734) is the first Food and Drug Administration (FDA) approved direct-acting antiviral for the treatment of SARS-CoV-2. RDV is a monophosphoramidate prodrug of the C-adenosine analog GS-441524 that acts by inhibiting RNA synthesis by the viral RNA-dependent RNA polymerase (*nsp12*-RdRp), and has been shown to be broadly active against multiple RNA viruses (*3*–*7*). Preferential incorporation of the triphosphate form of RDV (RDV-TP) over its natural adenosine-triphosphate (ATP) nucleotide counterpart results in inhibition of RNA synthesis through several mechanisms. Delayed chain termination may occur three nucleotides following RDV-TP incorporation due to a clash of the RDV-monophosphate (MP) 1’-cyano with S861 in the RdRp RNA exit channel, thereby preventing further enzyme translocation (*8*–*11*). However, increases in nucleoside-triphosphate (NTP) concentrations can overcome this obstacle and RNA synthesis can continue, resulting in RNA strands with incorporated RDV-monophosphate (RDV-MP) residues. In this setting, template-dependent inhibition of RNA synthesis occurs because of compromised incorporation of the complementary uridine-triphosphate (UTP) opposite RDV-MP (*12*–*14*).

The use of therapeutic RDV has been shown to improve disease outcomes and reduce viral loads in SARS-CoV-infected mice, in mice infected with chimeric SARS-CoV encoding the SARS-CoV-2 RdRp, in SARS-CoV-2 infected mice co-treated with therapeutic antibodies, and in infected rhesus macaques (*4*, *7*, *15*–*17*). Furthermore, RDV potently inhibits viral replication of both human endemic coronaviruses (CoVs) and bat CoVs in primary human lung cell cultures (*4*, *6*). A large double-blinded, randomized, placebo-controlled trial of intravenous RDV in adult patients hospitalized with coronavirus disease 2019 (COVID-19) demonstrated that RDV was superior to placebo in shortening time to recovery (*18*, *19*), and recent data suggests 50% increased survival rates if given early in infection (*20*). In addition, a 3-day early treatment course of RDV reduced the hospitalization of high-risk COVID-19 patients by 87% compared to placebo (*21*). However, a recent case report described the emergence of possible RDV resistance in an immunocompromised patient, underscoring the importance of further understanding pathways to RDV resistance (*22*). Little is known about the evolution, viral determinants, and specific mechanisms of SARS-CoV-2 resistance to RDV, limiting active surveillance for resistance-associated substitutions.

Here we report multiple pathways by which SARS-CoV-2 achieved varying degrees of resistance to RDV during serial passage in cell culture in the presence of GS-441524, the parent nucleoside of RDV, including multiple combinations of *nsp12*-RdRp amino acid substitutions (V166A, S759A, V792I, and C799F/R). Lineages containing S759A demonstrated 7-to-9-fold decreased sensitivity to RDV as shown by half-maximal effective concentration (EC_50_). Introduction of the SARS-CoV-2 co-selected S759A and V792I mutations at identical *nsp12*-RdRp residues in the betacoronavirus, murine hepatitis virus (MHV), conferred up to 38-fold increase in RDV EC_50_ but also incurred a replication defect compared to wild-type virus. Biochemical analyses of SARS-CoV-2 *nsp12*-RdRP with S759A and V792I mutations demonstrated distinct and complementary molecular mechanisms of RDV resistance. This study provides insights into potential evolutionary pathways leading to RDV resistance, identifies viral determinants and molecular mechanisms of RDV resistance, and forms the basis for surveillance for early indicators for potential RDV resistance.

## RESULTS

### SARS-CoV-2 acquires phenotypic resistance to RDV during passage with GS-441524.

**Fig. 1.**
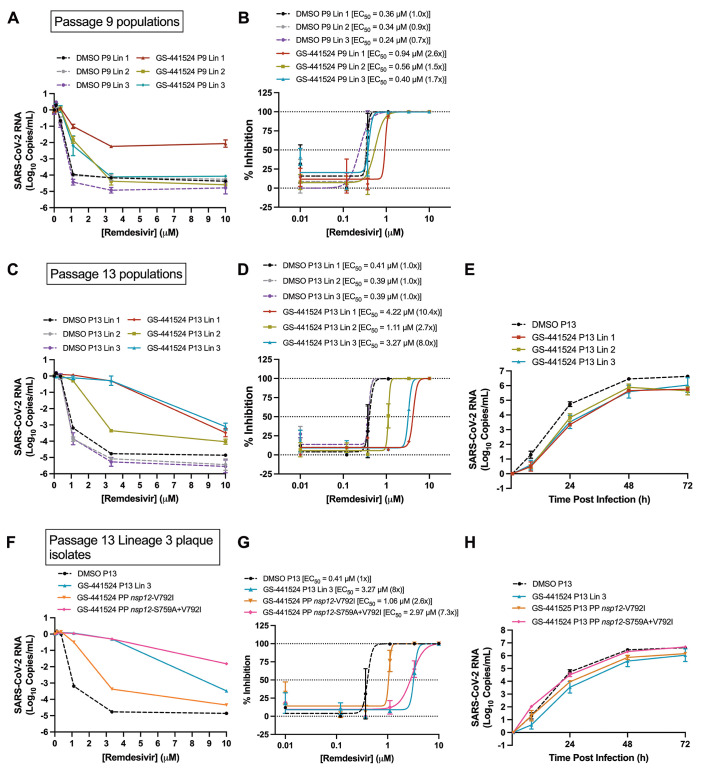
SARS-CoV-2 RDV resistance develops after serial passaging. SARS-CoV-2 was serially passaged in the presence and absence of GS-441524 in Vero-E6 cells in triplicate lineages. **(A)** Sensitivity of P9 lineages (Lin) to RDV in A549-hACE2 cells was determined by change in genome copy number. **(B)** Percent inhibition was calculated from genome copy number (A) and fold-change in EC_50_ compared to vehicle (DMSO)-passaged lineage 1 at P9. **(C)** Sensitivity of P13 lineages to RDV in A549-hACE2 cells was determined by change in genome copy number. **(D)** Percent inhibition calculated from genome copy number (C) and fold-change in EC_50_ compared to vehicle-passaged lineage 1 at P13. **(E)** Replication kinetics of P13 drug-passaged viral lineages are shown compared to vehicle-passaged lineage 1. **(F)** Sensitivity to RDV is shown for plaque-pick (PP) isolates from GS-441524-passaged lineage 3 and vehicle-passaged lineage 1 population viruses and input virus in A549-hACE2 cells, as determined by change in genome copy number. Plaque-picks (PP) from lineage 3 were isolated and expanded in presence of 1μM GS-441524. **(G)** Percent inhibition was calculated from raw genome copy number (F) and fold-change in EC_50_ compared to vehicle DMSO-passaged lineage 1. **(H)** Replication kinetics of plaque isolates tested in (F) and (G). Error bars indicate standard deviation.

We previously reported that RDV resistance mutations selected in MHV conferred resistance in SARS-CoV (*5*). To identify viral genetic pathways to RDV resistance in SARS-CoV-2, we passaged the WA-1 clinical isolate (MN985325) (*23*) in Vero E6 cells in the presence of dimethyl sulfoxide (DMSO) vehicle or GS-441524, which achieves higher concentrations of the active nucleoside triphosphate than the prodrug RDV in Vero E6 cells because it is metabolized more efficiently in this cell type (*7*). Virus was passaged in three independent and parallel series, resulting in three GS-441524-passaged lineages and three DMSO-passaged lineages (fig. S1). An increase in cytopathic effect (CPE) was observed in the all three GS-441524-passaged lineages between passages 10 and 13. To determine whether this shift represented selection for resistance, we tested the sensitivity of each drug- and DMSO-passaged lineage to RDV in the A549 human lung cell line expressing human angiotensin converting enzyme 2 (A549-hACE2), at passage 9 (P9) and passage 13 (P13) by quantifying the relative change in viral genome copy number in cell culture supernatant and determining EC_50_ concentrations (Fig. 1A to D, table S1). All three P9 GS-441524-passaged lineages were less sensitive to RDV than P9 DMSO lineages (Fig. 1A). GS-441524 lineage 1 appeared moderately less sensitive to RDV, with a 2.6-fold increase in EC_50_ at P9 (Fig. 1B, table S1). Lineage 1 RDV sensitivity decreased further by P13, with a 10.4-fold increase in EC_50_ (Fig. 1C and D, table S1). GS-441524 lineage 2 demonstrated minimal change in susceptibility to RDV at P9, with a 1.5-fold increase in EC_50_ compared to DMSO-passaged lineages (Fig. 1A and B, table S1) and its sensitivity further decreased modestly by P13, with a 2.7-fold increase in EC_50_ compared to DMSO-passaged lineages (Fig. 1C and D, table S1). At P9, GS-441524 lineage 3 demonstrated a 1.7-fold increase in EC_50_ compared to the DMSO-passaged lineages (Fig. 1B, table S1). Sensitivity of GS-441524 lineage 3 decreased further by P13, with an 8-fold increase in EC_50_ compared to DMSO-passaged lineage 1 (Fig. 1D, table S1). We next tested the replication of all the DMSO and GS-441524-passage lineages in the absence of RDV. Compared to the clear replication advantage observed in the presence of RDV, all three GS-441524-passaged P13 lineages demonstrated delayed and impaired replication with a 0.5 to 1 log decrease in peak titer compared to DMSO-passaged control virus in the absence of drug (Fig. 1E). Thus, selection for phenotypic resistance conferred a replication defect in all three lineages.

### Identification of candidate resistance mutations in *nsp12*-RdRp

To identify candidate resistance mutations, we performed short-read Illumina poly(A) RNA sequencing (RNA-seq) on RNA purified from infected cell monolayers of all six lineages at passages 0 (input), 6, 9, and 13. (Table 1, data file S1). Numerous low frequency nucleotide mutations (0.01 to 5%) were detected in all six lineages at P13 (data file S1). Of the non-synonymous (NS) mutations present at greater than 15% frequency, mutations in spike were present in both DMSO- and drug-passaged lineages and likely represent cell culture adaptation (data file S1). In contrast, six NS *nsp12*-RdRp mutations were detected with greater than 15% frequency in GS-441524-passaged lineages, but not in any of the DMSO-passaged lineages (Table 1). By P13, these *nsp12-*RdRp NS mutations were dominant (>50%) in GS-441524-passaged populations. GS-441524 lineage 1 encoded NS *nsp12*-RdRp mutations V166A (92%), N198S (97%), S759A (62%) and C799F (38%); lineage 2 encoded only C799R; and lineage 3 encoded S759A (99%) and V792I (99%) (Table 1, data file S1). GS-441524 lineage 1 and 3 populations were more resistant than GS-441524 lineage 2 based on both extent of CPE and the increased RDV EC_50_ (Fig. 1C and D). Of the selected substitutions, only S759A was detected in the original SARS-CoV-2 WA-1 P5 stock virus population at 0.64%. The S759A was not detected at any frequency in any of the lineages passaged in the DMSO vehicle (Table 1, data file S1), suggesting a lack of positive selection in absence of RDV. Overall, these results identified distinct combinations of a limited number of *nsp12*-RdRp NS mutations associated with independent RDV-resistant lineages.

**Table 1.** *nsp12* non-synonymous mutations present at greater than 15% of populations of serially passaged SARS-CoV-2. SARS-CoV-2 was passaged 13 times in increasing concentrations of GS-441524 or vehicle (DMSO) in three lineages each. Values represent percent of mutations detected by RNA-seq at passage 13 in RNA extracted from infected cell monolayers. PP: sub-lineages isolated from plaque picks. Refer to data file S1 for complete dataset.

	***nsp12***					
*Genome Position*	13937	14033	15715	15814	15835	15836
*Amino Acid Changes*	**V166A**	**N198S**	**S759A**	**V792I**	**C799R**	**C799F**
Input Virus (SARS-CoV-2 WA-1 P5 Stock)	-	-	0.64%	-	-	-
DMSO P13 Lineage 1						
DMSO P13 Lineage 2						
DMSO P13 Lineage 3						
GS-441524 P13 Lineage 1	92.32%	97.57%	61.72%			38.31%
GS-441524 P13 Lineage 2					86.27%	
GS-441524 P13 Lineage 3			99.27%	98.82%		
PP *nsp12*-V792I				99.96%		
PP *nsp12*-S759A+V792I			99.72%	99.97%		

To look for presence of the identified in vitro GS-441524-selected *nsp12* mutations in circulating clinical isolates we analyzed the consensus sequences of >8 million clinical isolates of SARS-CoV-2 submitted to the GISAID Database (*24*) prior to March 18, 2022 (table S2). In the absence of RDV selection, the S759A mutation was identified in submitted consensus sequences, including the Delta variant only in a single isolate whereas the other *nsp12* mutations were detected at a frequency less than 0.01% (N=969). A separate analysis of the Delta and recently emerged Omicron variant sequences was performed. S759A was not observed and other substitutions were detected at comparable or lower frequency than the full global dataset. A clear limitation of this dataset is that the details of isolation and raw sequence data are not available. Consensus sequences most likely represent nucleotides present at >50% and that any single nucleotide polymorphisms present <50% in the population would not be represented in this analysis. For example, we detected the S759A substitution at 0.64% in the expanded clinical WA-1 isolate received from the Centers for Disease Control and Prevention (CDC), which may have importance for its eventual selection. Similarly, in the GS-441524 lineage 1 passage 13, C799F was present at 38%, a frequency that would not be reported in consensus sequence (Table 1). Despite this limitation, the analysis can allow us to conclude that, in the absence of RDV-selective pressure, the in vitro identified *nsp12* mutations were not present as dominant variants or propagated in circulating SARS-CoV-2 variants, including Delta and Omicron. This supports our hypothesis that the identified *nsp12* mutations likely were associated with GS-441524 selective pressure, a hypothesis we next tested.

### *nsp12*-RdRp S759A and -V792I are associated with in vitro RDV resistance

The S759A residue substitution was selected in GS-441524 lineages 1 and 3, which demonstrated the most CPE and EC_50_ increase of GS-441524-passaged lineages (Fig. 1, fig. S1). In both lineages, the S759A substitution emerged as a dominant change in the population in combination with at least one other substitution; GS-441524 lineage 3 contained only S759A and V792I. To define the contribution of S759A and V792I to RDV resistance in the context of adapted infectious virus, we plaque-picked (PP), expanded, and sequenced sub-lineages derived from GS-441524-passaged lineage 3 (Table 1, data file S1). We isolated a sub-lineage containing V792I alone and another sub-lineage containing S759A in combination with V792I. Importantly, in these two sub-lineages, no other NS mutations were detected at greater than 5% elsewhere in replicase genes (data file S1). In RDV sensitivity assays, the S759A+V792I-containing sub-lineage was indistinguishable from the GS-441524-passaged lineage 3 population at RDV concentrations up to 3.3μM. A 7.3-fold increase in EC_50_ was observed over the DMSO-passaged control as compared to an 8-fold increase in EC_50_ for the GS-441524 lineage 3 P13 population virus (Fig. 1F and G, table S1). Thus, the S759A+V792I plaque-picked sub-lineage recapitulated resistance phenotype of the GS-441524 lineage 3 P13 population. In contrast, the V792I-containing sub-lineage displayed a low degree of resistance corresponding to a 2.6-fold increase in EC_50_ compared to the DMSO-passaged virus. Finally, in the absence of RDV, the V792I and S759A+V792I plaque-picked sub-lineages demonstrated replication kinetics similar to the DMSO-passaged control virus rather than to the GS-441524 lineage 3 population virus (Fig. 1H), suggesting differential replication efficiency among subpopulations of GS-441524-passaged virus.

### Selection and emergence of candidate resistance mutations and combinations.

**Fig. 2.**
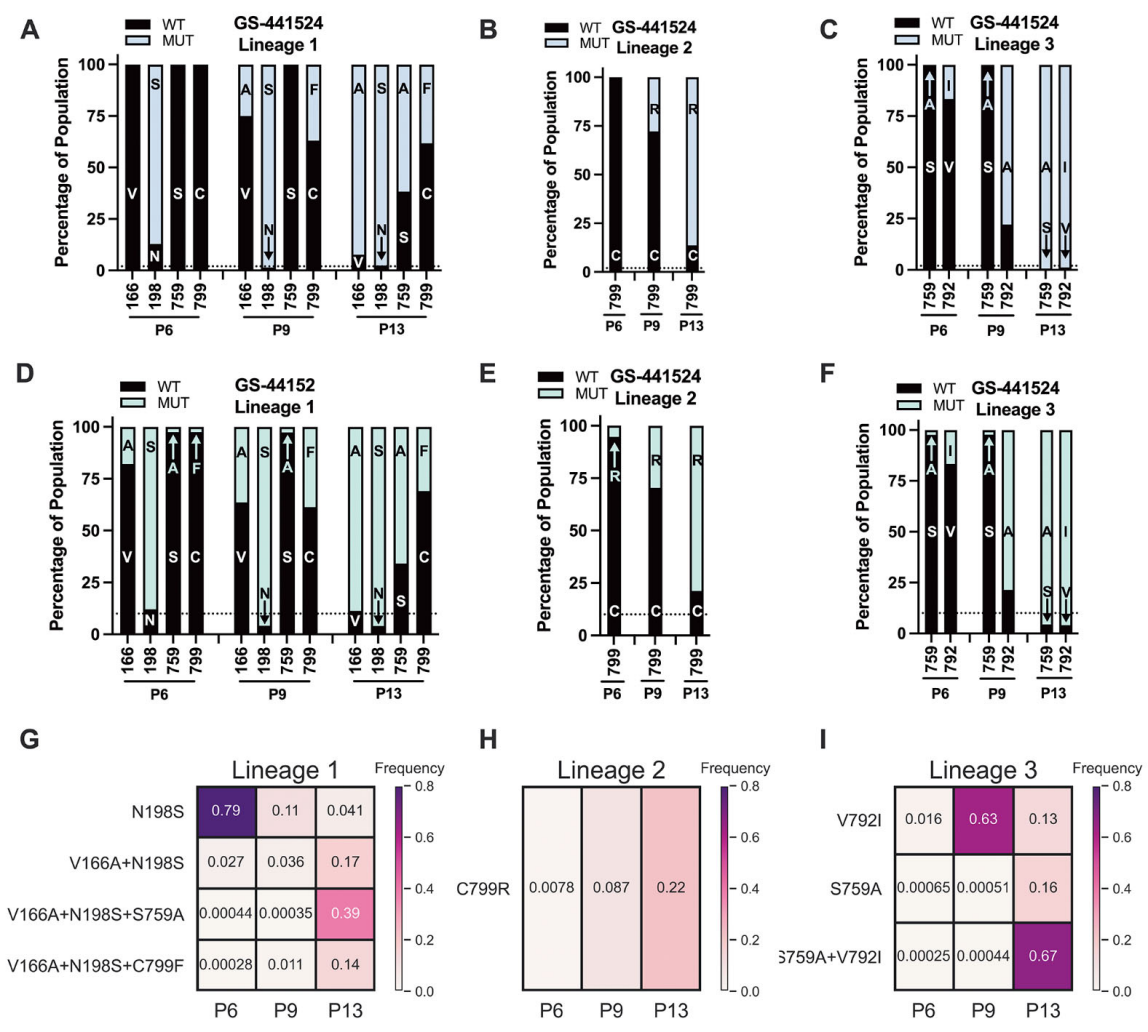
Evolution and intramolecular linkage of *nsp12* mutations. SARS-CoV-2 was passaged 13 times in increasing concentrations of GS-441524 in 3 lineages. RNA from infected cell monolayers was subjected to Illumina RNA sequencing and Oxford nanopore MinION sequencing. **(A to C)** RNA-seq was used to measure the percent of *nsp12* mutations in lineages 1 (A), 2 (B), and 3 (C). **(D to F)** Nanopore amplicon sequencing was used to measure percent of *nsp12* mutations (MUT) in lineages 1 (D), 2 (E), and 3 (F) relative to wild-type (WT) *nsp12*. **(G to I)** The frequency of single and combined sets of *nsp12* mutations is shown for in single viral genomes in lineages 1 (G), 2 (H), and 3 (I). Variants were mapped according to their genomic position and frequency, expressed as a percentage of the total reads mapped to that position.

To define viral genetic progression toward resistance, we quantified the relative frequency of RDV resistance-associated *nsp12* mutations in the GS-441524 lineages 1, 2, and 3 at P6, P9, and P13 by RNA-seq (Fig. 2A to C) and direct nanopore MinION sequencing of DNA amplicons spanning *nsp12* coding domain (Fig. 2D to F). The data results between the two methods were consistent in the emergence and patterns. In lineage 1, N198F was present in the population at greater than 87% by P6 and thereafter, whereas V166A, S759A, and C799F were less abundant at P6 but were prominent by P13 (Fig. 2A and D). In lineage 2, only C799R was detected at any passage by RNA-seq or nanopore amplicon sequencing (Fig. 2B and E). In lineage 3, V792I became dominant in the P9 population, whereas S759A was detected at about 2.5% by nanopore amplicon sequencing at P6 and P9, but by P13 was greater than 65% (Fig. 2C and F). In both lineage 1 and 3, emergence of S759A correlated with an increase in EC_50_ (2.6-to-10.4-fold in lineage 1; 1.7-to-8.0-fold in lineage 3) (Fig. 1, Fig. 2, table S1).

To determine the frequency with which resistance-associated mutations were present individually or in haplotypic linkages, we employed long-read nanopore (MinION) sequencing across full-length *nsp12* amplicons and developed the bioinformatics pipeline, *MutALink*, to quantify the absolute abundance of each mutation alone or in combination with other mutations that occurred at greater than 15% frequency by P13 (Fig. 2 G to I). Of the *nsp12*-RdRp amino acid substitutions identified in lineage 1, the abundance of combined V166A+N198S+S759A underwent the greatest increase with increasing drug concentration (Fig. 2G). In lineage 2, the C799R mutation had no detected linkage to another mutation (Fig. 2H). In lineage 3, the abundance of combined S759A+V792I demonstrated the greatest increase with increasing drug concentration (39%) (Fig. 2I). Thus, S759A predominantly existed in combination with either N198S+V166A (lineage 1) or V792I (lineage 3).

### Structural modeling of S759A predicts altered RDV interactions.

**Fig. 3.**
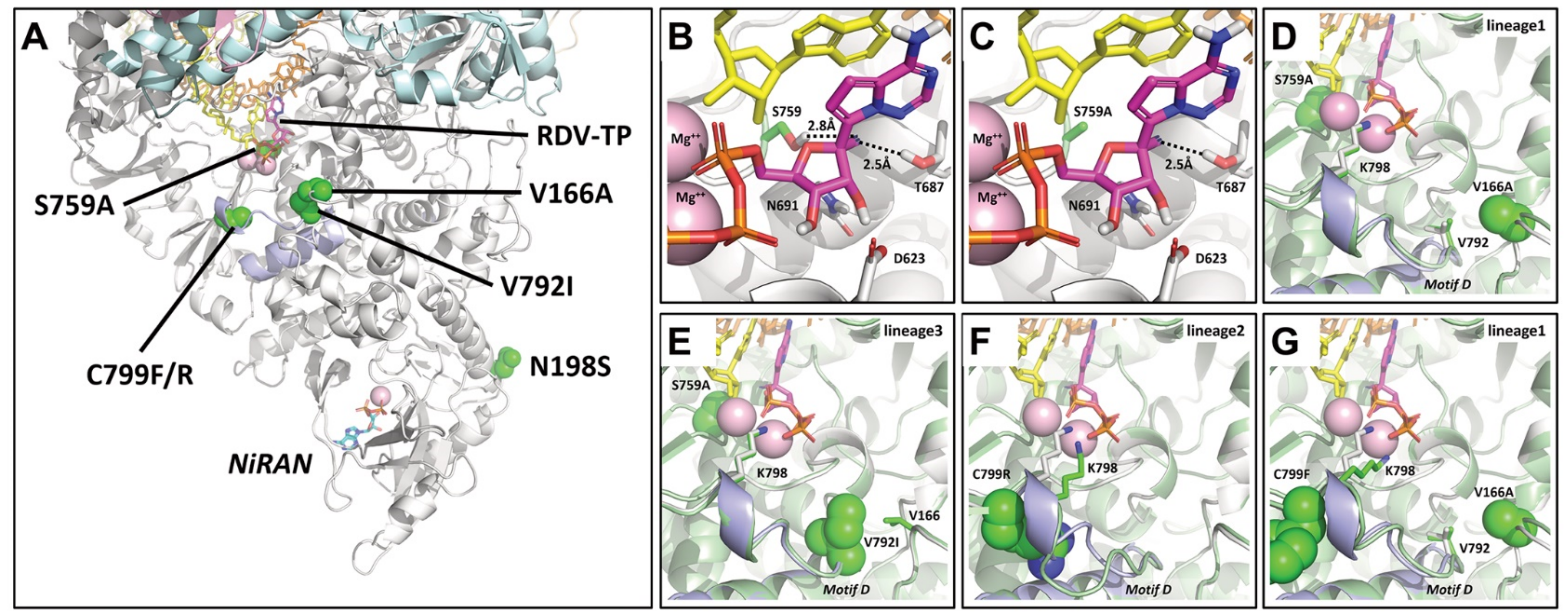
**Structural modelling predictions of identified *nsp12*-RdRp mutations. (A)** Observed *nsp12* amino acid substitutions were mapped on a model of the SARS-CoV-2/RDV-TP pre-incorporation complex. nsp12 is shown in white, nsp7 in pink, nsp8 in cyan, the primer strand in yellow, the template strand in orange, RDV-TP in magenta, and mutations in green. S759A is in the active site, whereas V166A, V792A and C799F/R are adjacent to the active site, clustered around motif D (in blue). Details of the RDV-TP pre-incorporation model are shown, highlighting the polar residues that interact with the 2’OH and the 1’CN. (**B**) S759 is seen to be in close contact with the 1’CN, forming a favorable interaction. (**C**) Substitution of the serine with an alanine increases the distance between residue 759 and the 1’CN, resulting in a loss of favorable interaction. N198S does not appear to impact either the NiRAN or Pol sites. **(D)** A model of the lineage 1 mutations V166A and S759A (green) overlaid on the wild-type structure (white) is shown. V166A is in direct contact with V792 and may impact the dynamics of motif D. **(E)** A model of the similar lineage 3 mutations V792I and S759A (green) overlaid on the wild-type structure (white) is shown. **(F)** A model of the lineage 2 mutation C799R (green) overlaid on the wild-type structure (white) is shown. The mutation is predicted to alter the conformation of motif D, impacting how K798 interacts with the substrate γ-phosphate. **(G)** A model of the lineage 1 mutations V166A and C799F is shown, which are also observed to alter the conformation of motif D and the position of K798.

The potential of RDV resistance-associated *nsp12* mutations to accommodate the RDV-TP substrate was evaluated using a structural model of the pre-incorporation state in which the NTP substrate is base paired to the template and coordinated with two Mg^++^ ions in the active site (Fig. 3). The model was generated based on the SARS-CoV-2 RdRp cryo-EM structure 6XEZ (*25*), but was influenced by several other polymerase structures (*26*–*28*). From this model, we determined that the amino acid substitutions which arose during serial drug passage were clustered in three general locations: S759A was located in the RdRp catalytic S_759_DD motif C, in close proximity to the incoming NTP; V166A, V792I and C799F/R were located on or adjacent to motif D, a common structural element of polymerases important to the dynamics of NTP incorporation; and N198S was located on the protein surface behind the Nidovirus RdRp associated nucleotidyl transferase domain (NiRAN) site, a position having no known or predicted role in RNA synthesis (Fig. 3A). Focusing on the active site, several polar residues form a pocket containing T687, A688 and N691 on motif B and S759 on motif C that could easily accommodate the RDV-TP 1’-cyano group (Fig. 3B). Some variation in the sidechain conformations of T687 and S759 was observed across the available cryo-EM structures and may be dependent on the state of RNA and substrate binding. A computational assessment of these conformations within the RDV-TP pre-incorporation model suggested that a state which oriented the hydroxyls toward the RDV-TP 1’-cyano was optimal. The resulting hydrogen bonds were weak (2.5 Å for T287 and 2.8 Å for S759), but this overall favorable interaction suggested a potential advantage of RDV-TP over natural substrates. Modeling the S759A mutation showed a loss of one of these hydrogen bonds (Fig. 3C). From that we predicted decreased binding affinity of RDV-TP in the pre-incorporation complex relative to ATP. The effect of other observed mutations was more difficult to predict structurally. A conformational search of the RdRp loops 163 to 168 and 790 to 800 for both wild-type and mutant amino acids predicted only modest shifts for V166A+S759A and S759A+V792I (Fig. 3D and E). In contrast, the C799R and V166A+C799F mutations predicted clear changes in the conformation of motif D (Fig. 3F and G). As motif D is thought to be important to the closing of the polymerase active site once the NTP is positioned for incorporation (*29*), any mutation affecting dynamics of this loop could potentially impact incorporation rates.

### SARS-CoV-2 *nsp12*-RdRp S759A and V792I mutation homologs confer RDV resistance in recombinant murine hepatitis virus.

**Fig. 4.**
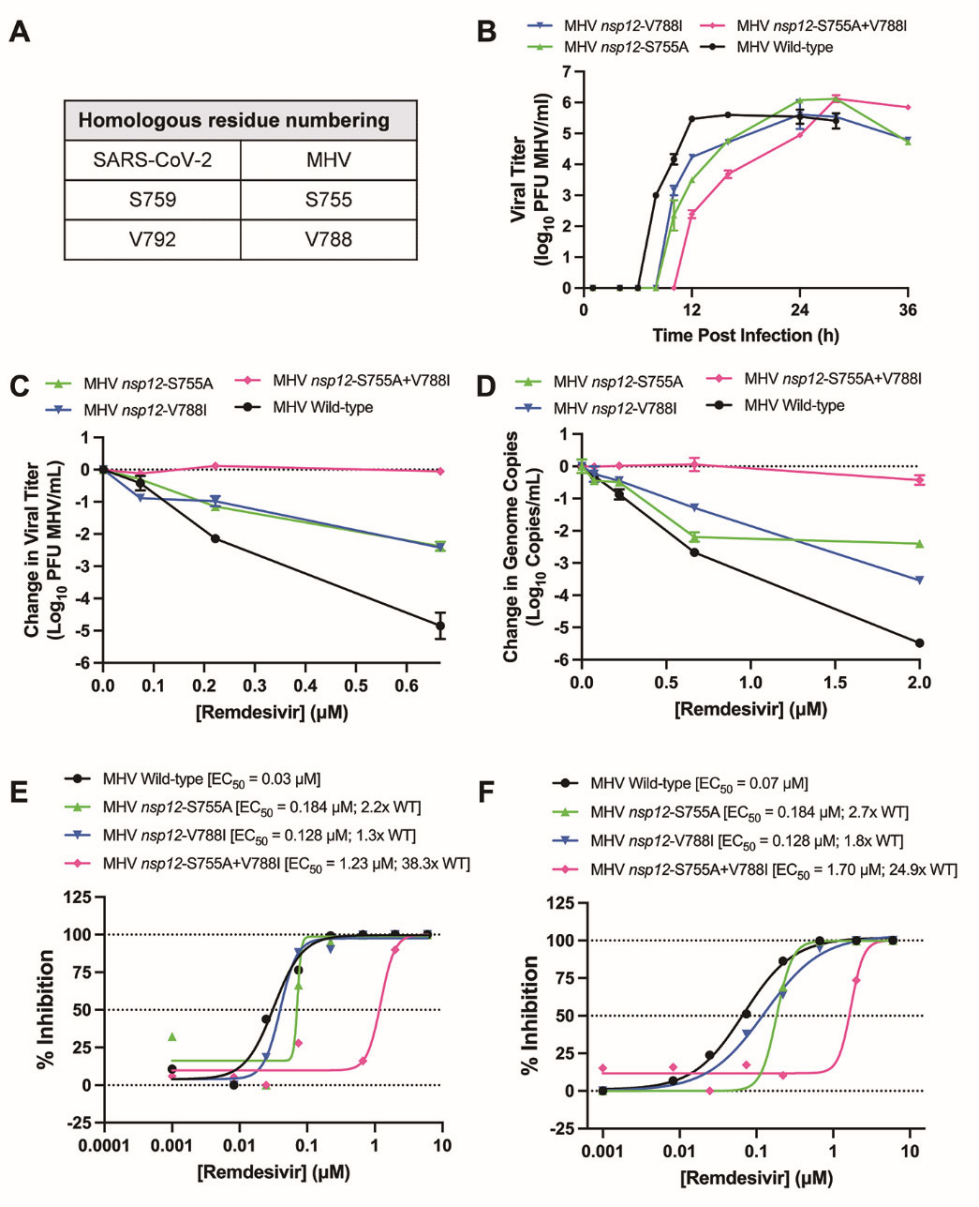
**SARS-CoV-2 resistance mutations confer RDV resistance in MHV. (A)** Candidate resistance mutations identified in SARS-CoV-2 were engineered at conserved homologous positions in the MHV infectious clone. Wild-type MHV and mutant viruses were tested against RDV in murine delayed brain tumor (DBT) cells. **(B)** MHV replication kinetics. **(C)** Change in infectious viral titers as measured by plaque assay. Changes are shown relative to DMSO control treatment. **(D)** Change in MHV genome copy number as measured using qRT-PCR. **(E)** Percent inhibition and EC_50_ values calculated using infectious virus titers from (B). **(F)** Percent inhibition and EC_50_ values calculated using genome copy numbers from (C).

In all subsequent experiments we targeted S759A and V792I for genetic and biochemical analysis because they were associated with the most increase in measured resistance, they could be isolated alone (V792I) and together (S759A+V792I) in virus clones, and S759A, located in the *nsp12*-RdRp LS_759_DD active site motif, modeled a plausible change in interaction with RNA. To directly test the capacity of S759A and V792I substitutions to mediate RDV resistance in isogenic virus backgrounds devoid of other mutations, we engineered individual S-to-A and V-to-I changes at the aligned identical and structurally orthologous S755 and V788 residues in the *nsp12*-RdRp of MHV (Fig. 4A). Viable MHV mutants encoding S755A, V788I, or S755A+V788I were compared with wild-type MHV in replication assays in the absence of RDV. Although all mutant viruses ultimately attained peak titers similar to wild-type, there were defects in mutant virus replication kinetics (Fig. 4B). The MHV S755A and V788I mutants demonstrated a 2-hour delay to exponential replication compared to wild-type MHV and a 12-hour delay to peak titer. The S755A+V788I double mutant conferred a more protracted 4-hour delay to exponential replication and 16-hour delay to peak titer. Analysis of sensitivity to RDV demonstrated that MHV mutants were less sensitive to RDV than wild-type MHV based on reduction in infectious viral titer (Fig. 4C) and genome copy number (Fig. 4D). MHV mutants encoding S755A or V788I alone demonstrated small decreases in sensitivity to RDV compared to wild-type MHV by EC_50_ calculation. For S755A, there was a 2.2-fold increase in EC_50_ by infectious virus titer (plaque assay) (Fig. 4E) and 2.7-fold increase based on RNA genome copy number (Fig. 4F). Corresponding values for V788I were 1.3-fold and 1.8-fold increase in EC_50_, respectively (Fig. 4E and F). In contrast, the combined S755A+V788I mutant demonstrated a of 38.3-fold increase in EC_50_ compared to wild-type based on infectious viral titer and 24.9-fold increase in EC_50_ based on genome copy number (Fig. 4E and F). Further, the combination S755A+V788I mutant demonstrated complete resistance to RDV at a concentration of RDV that caused greater than 99% inhibition of wild-type MHV (0.6 mM) (Fig. 4E and F). Thus, GS-441524-selected SARS-CoV-2 S759A and V792I-associated phenotypes were transferable to MHV and mediated RDV resistance in the MHV genetic background. The results also demonstrated that the substitutions, when tested in isolation or in combination, impaired virus replication efficiency in the absence of RDV, consistent with our previous study demonstrating that RDV resistance selection in MHV was achieved at the expense of viral fitness (*5*).

### SARS-CoV-2 *nsp12*-RdRp S759A and V792I substitutions mediate RDV resistance by independent and complementary biochemical mechanisms

**Fig. 5.**
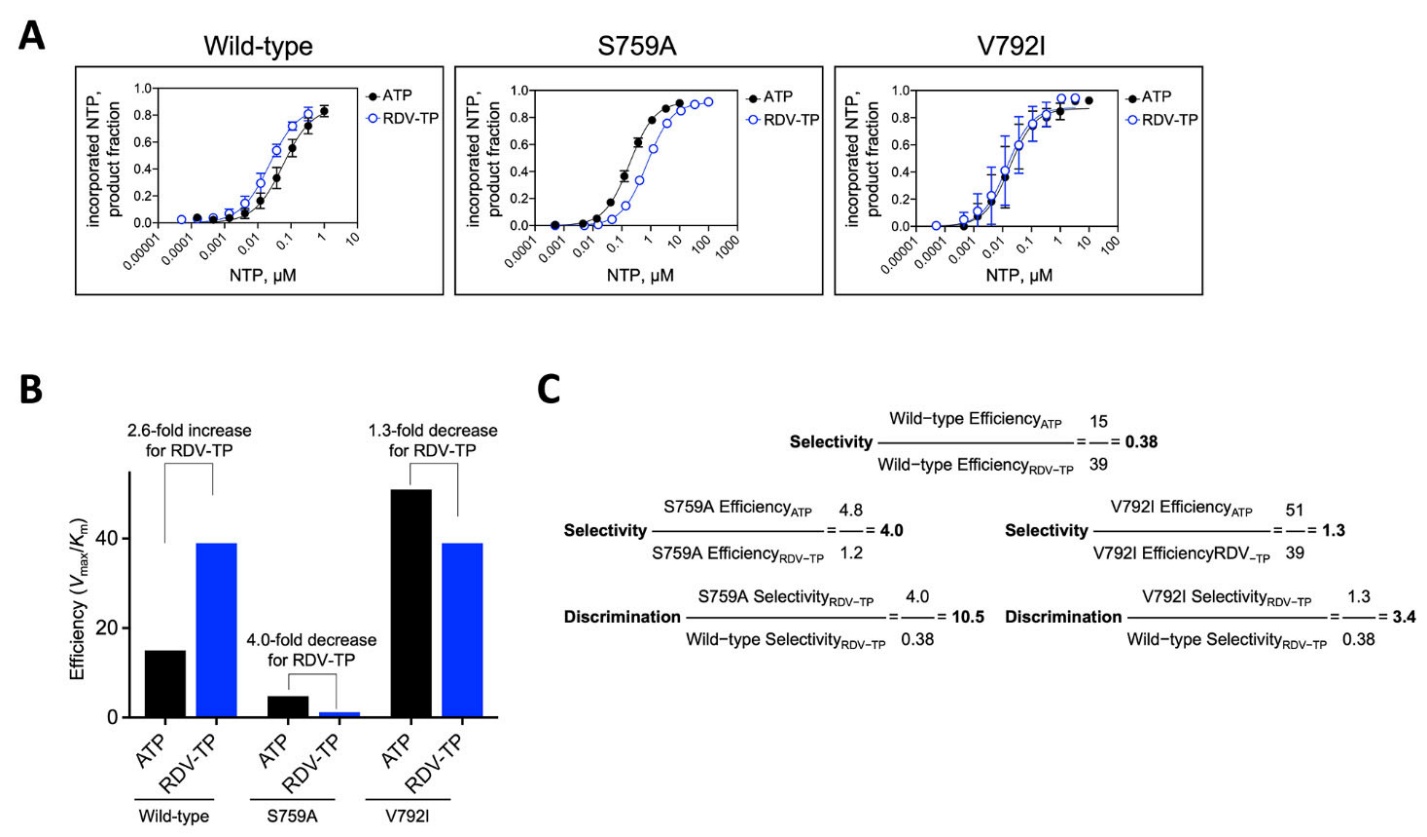
RDV-TP is differentially incorporated by wild-type, S759A, and V792I mutant SARS-CoV-2 RdRp complexes. (**A**) Graphical representation of ATP or RDV-TP single nucleotide incorporation during RNA synthesis as a function of their respective concentrations shown in fig. S2. Best fit lines illustrate fitting of the data points to Michaelis-Menten kinetics function using GraphPad Prism 7.0. Error bars illustrate the standard deviation of the data. All data represent at least three independent experiments. (**B**) The efficiencies of incorporation (ATP and RDV-TP) and selectivity (ATP over RDV-TP) of the mutant enzymes were quantified and corrected for differences in ATP incorporation. (**C**) Selectivity and discrimination values were calculated for RDV-TP across mutant enzymes.

We next sought to define the mechanisms of S759A and V792I-mediated resistance. For biochemical analysis, we expressed and purified SARS-CoV-2 wild-type, S759A, or V792I RdRp complexes consisting of *nsp7*, *nsp8*, and *nsp12*, using approaches previously described (*8*). A key characteristic of RDV-TP as an inhibitor of wild-type SARS-CoV-2 is that it is incorporated with higher efficiency than its natural ATP counterpart by the viral RdRp (*8*, *30*). To determine if a change in selective substrate usage could explain the effect of S759A and V792I, we determined the efficiency of incorporation of ATP over RDV-TP by measuring kinetic parameters for single nucleotide incorporation events under steady-state conditions (Fig. 5A, fig. S2, and Table 2). *V*_max_ and *K*_m_ are Michaelis-Menten parameters that reflect the maximum velocity of nucleotide incorporation and the concentration of nucleotide substrate at ½ *V*_max_, respectively. The ratio of *V*_max_ over *K*_m_ is the measure of efficiency for nucleotide incorporation. ATP and RDV-TP incorporation were monitored with a model primer/template that adequately mimic RNA synthesis using wild-type RdRp and the S759A and V792I mutants (fig. S2). Selectivity is defined by the ratio of single nucleotide incorporation efficiencies of ATP over RDV-TP (Fig. 5B and C). Consistent with previous reports (*8*, *30*), the selectivity measured with the wild-type enzyme was 0.38 indicating that RDV-TP was preferred over ATP (Table 2). In contrast, the selectivity value for the S759A mutant was 4.0, demonstrating a preferred use of ATP over RDV-TP. This shift was driven primarily by a marked reduction in the use of RDV-TP as a substrate (33-fold decrease in *V*_max_/*K*_m_). The efficiency of incorporation of ATP also was compromised with S759A to a lesser degree (3.1-fold decrease in *V*_max_/*K*_m_). When corrected for the difference in ATP usage, the S759A mutant showed a 10.5 fold reduced use of RDV-TP as substrate relative to the use of ATP. In contrast, the RdRp expressing V792I demonstrated a 3.4-fold increased capacity to incorporate ATP, whereas the increase in RDV-TP substrate usage was less pronounced. This resulted in a selectivity value of about 1.3 only a 3.4-fold change compared with the wild-type RdRp (Table 2). Thus, the S759A mutant discriminated against the inhibitor RDV-TP at the level of nucleotide incorporation much more effectively than V792I.

**Table 2.** Selectivity values against RDV-TP incorporation by SARS-CoV-2 wild-type and a set of mutants containing single amino acid substitution at residue S759.

**RdRp**	**V_max_ *^a^***		**K_m_ *^b^***		**V_max_ / K_m_**		**Selectivity *^c^***	**Discrimination *^d^***
	**ATP**	**RDV-TP**	**ATP**	**RDV-TP**	**ATP**	**RDV-TP**		
**Wild-type** n=10 ***^e^***	0.86 ±0.016 ***^f^***	0.86 ±0.015	0.058 ±0.0041	0.022 ±0.0015	15	39	0.38	Ref. ***^g^***
**S759A** n=3	0.92 ±0.008	0.91 ±0.006	0.19 ±0.0075	0.74 ±0.025	4.8	1.2	4.0	10.5
**V792I** n=3	0.87 ±0.039	0.90 ±0.031	0.017 ±0.0040	0.023 ±0.0035	51	39	1.3	3.4

***^a^*** *V*_max_ is a Michaelis–Menten parameter reflecting the maximal velocity of nucleotide incorporation; reported as a raw value of product fraction of the incorporated nucleotide. ***^b^*** *K*_m_ is a Michaelis–Menten parameter reflecting the concentration (μM) of the nucleotide substrate at which the velocity of nucleotide incorporation is half of *V*_max._ ***^c^*** Selectivity of a viral RNA polymerase for a nucleotide substrate analog is calculated as the ratio of the *V*_max_/*K*_m_ values for NTP and NTP analog, respectively; as such, it is a unitless value. ***^d^*** The discrimination index is calculated as a ratio of selectivity of the mutant over wild-type. ***^e^*** All reported values have been calculated on the basis of a 9-data point experiment repeated the indicated number of times (n).

***^f^*** Standard error associated with the fit. ***^g^*** Reference value used to determine discrimination by mutant enzymes.

Next, we used a second biochemical assay to assess both the effect of changes in selective incorporation of ATP versus RDV-TP as well as the inhibitory effects of the incorporated RDV-MP on primer extension. We utilized a polyU template and increasing RDV-TP concentrations to enhance the resistant phenotypes by allowing multiple incorporations of RDV-TP, which could potentially magnify effects of the mutations (fig. S3A). In this in vitro model for wild-type RdRp, initially increasing RDV-TP concentration resulted in increased chain-termination (fig. S3B). However, further increases in RDV-TP concentration resulted in an increase in full-length product due to efficient RDV-TP substrate incorporation on the polyU template which overcame delayed chain termination. The V792I substitution showed a very similar pattern to wild-type, with only marginal increases in full-length product formation. Conversely, both the S759A and S759A+V792I mutants demonstrated delayed chain-termination only at higher concentrations compared to wild-type, and no rebound in full-length product formation was observed as RDV-TP concentrations increased. S759A and S759A+V792I were indistinguishable in this assay, suggesting that the phenotype is driven by S759A and the reduced usage of RDV-TP as a substrate. In contrast, the V792I substitution alone did not demonstrate an effect on selective incorporation of RDV-TP, delayed chain-termination, or overcoming delayed chain-termination (Table 2, Fig. 5, and fig. S3B). This result indicated that the contribution of V792I to RDV resistance is likely based on a different mechanism.

**Fig. 6.**
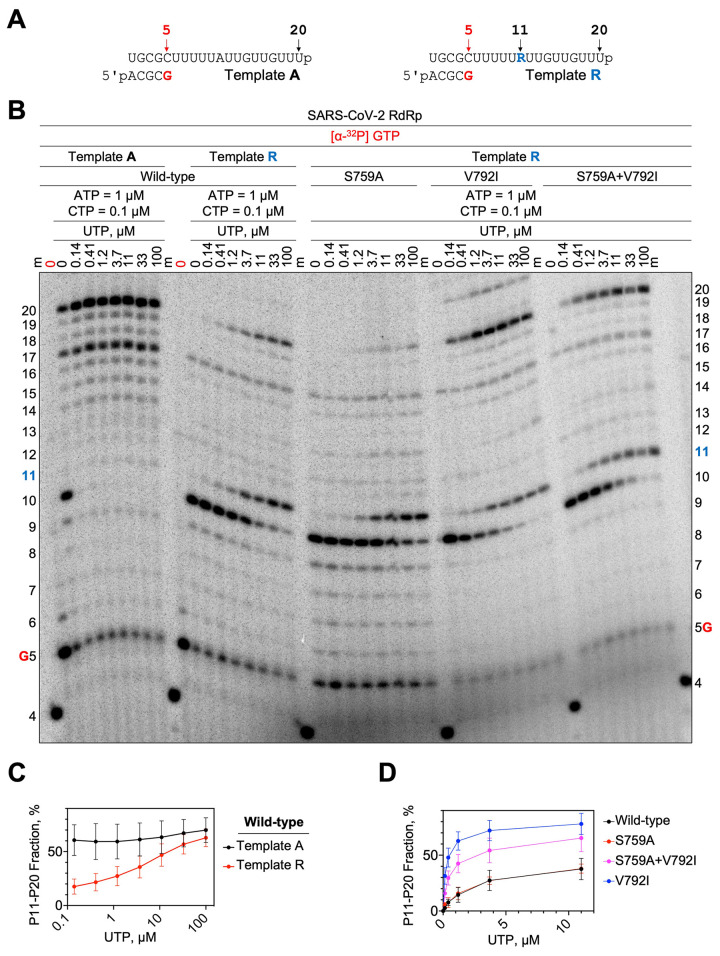
**RNA synthesis differs between SARS-CoV-2 wild-type and mutant S759A, V792I, and S759A+V792I RdRp complexes. (A)** The RNA primer/template sequences used are shown. **(B)** RDV-MP is embedded at position 11 in the template R strand while AMP is in the same position on the template A strand. RNA products were synthesized by the wild-type or mutant SARS-CoV-2 RdRps in a reaction mixture containing the primer/template pair, MgCl_2_, and indicated NTP concentrations. G (red) indicates the incorporation of [α-^32^P] GTP at position 5 and 4 indicates the migration pattern of 5′-^32^P-labeled 4-nt primer is used as a size marker. The 0 point in red indicates a reaction where [α-^32^P] GTP was the only NTP present to control for contaminating NTPs in the template preparations. **(C and D)** The fraction of RNA synthesis beyond position 11 with respect to total RNA products formed was quantified. **(C)** The comparison of reactions using template A and template R with wild-type RdRp and increasing concentrations of UTP is shown. **(D)** Comparisons of RNA synthesis by wild-type and mutant enzymes are shown. Data corresponding to UTP = 33 and 100 μM were excluded to focus on the differences in the lower concentration range.

We previously reported that the MHV-V553L RDV resistance mutation, when tested as the homologous V557L substitution in a SARS-CoV-2 biochemical system, conferred a low degree of RDV resistance by improving incorporation of UTP opposite the RDV-MP in the template and thereby reducing template-dependent inhibition (*12*). To test a potential effect of V792I, S759A, and S759A+V792I on template-dependent inhibition, we prepared wild-type template-A in which a single adenosine-monophosphate (AMP) was embedded, and template-R in which a single RDV-MP was embedded (Fig. 6A). All enzymes were equilibrated so that the same amount of product was present in the absence of UTP (Fig. 6B, lane “0”, product 10). The S759A mutant behaved almost identically to wild-type in this reaction, whereas V792I alone or S759A+V792I together lowered the UTP concentration needed to overcome template-dependent inhibition (Fig. 6C and D). Thus, distinct and complementary mechanisms of resistance were associated with S759A and V792I, and the two residue substitutions combined provided an advantage to RNA synthesis in the presence of RDV.

## DISCUSSION

We here show that SARS-CoV-2 is capable of evolving reduced susceptibility to GS-441524/RDV through substitutions in the *nsp12*-RdRp at, or in close proximity to, the RdRp S_759_DD active motif. Distinct sets of mutations within the RdRp arose in three separate lineages with differing degrees of population resistance, with lineage 3 co-evolving S759A and V792I substitutions that together in plaque isolates demonstrated the greatest RDV resistance. Introduction of the substitutions at homologous positions in the structurally conserved MHV *nsp12*-RdRp (S755A and V788I) confirmed the resistance phenotype and its transferability across divergent CoVs. Biochemical kinetic studies of the mutations in the expressed RdRp complex consisting of *nsp7*, *8* and *12* demonstrated that S759A improved RdRp discrimination against incorporation of RDV-TP, whereas V792I reduced template-dependent inhibition of RNA synthesis mediated by incorporated RMP, thereby complementing the effects of S759A. These results provide a mechanistic explanation for the co-selection and emergence of these mutations.

Although RdRp mutations previously have been reported in MHV (*5*) and SARS-CoV-2 (*31*, *32*) associated with RDV resistance, this report identifies an amino acid substitution in a CoV *nsp12*-RdRp S_759_DD catalytic motif that mediates a high magnitude of RDV. Notably, mutations are well-known at the structurally equivalent HIV-1 reverse transcriptase (RT) YM_184_DD active motif that confer resistance to nucleoside analogs (*33*). The HIV-1 RT YM_184_DD motif is relatively conserved among RT enzymes and M184V or M184I within this region has an effect on RT catalytic activity and may confer increased resistance (>100-fold) to lamivudine (Epivir) and emtricitabine (Emtriva) (*32*, *34*, *35*). In our study, replacing the conserved S759 in the S_759_DD motif of the RdRp with an alanine resulted in decreased sensitivity to RDV, yet exerted diminished impact compared to similar changes in the HIV-1 RT. Nevertheless, these data pinpoint S759A—the product of a single nucleotide change and tolerated amino acid substitution in the RdRp—as a likely key determinant of RDV resistance. Finally, it remains to be determined if our in vitro-selected S759A, V792I, or other mutations emerge in vivo under selection. Here, the HIV-1 example is potentially informative as the RT M184V/I substitutions first identified in vitro have repeatedly been confirmed to be selected in vivo (*36*).

The results of this study, along with our published biochemical and genetic studies, suggest that there are multiple potential genetic pathways to SARS-CoV-2 RDV resistance. These pathways may evolve both common and unique determinants within and across divergent CoVs. There remain many questions to pursue in understanding the relationship of RDV with the uniquely complex CoV multi-protein replicase, and the likely equally complex pathways to resistance in vitro and in vivo. Our previously reported MHV *nsp12*-RdRp RDV resistance substitutions F476L and V553L (*5*) were not detected at SARS-CoV-2 homologous F480 and V557 residues in any of the three GS-441524-passaged lineages in this study. However, our previous biochemical studies demonstrated that the SARS-CoV-2 V557L change did reduce template-dependent inhibition of RNA synthesis (*12*). In contrast, although the SARS-CoV-2 RDV resistance mutations S759A and V792I were not identified during MHV passage, introduction of the homologous substitutions in recombinant MHV mutants yielded clear reduced susceptibility to RDV alone and combined. The difference in the magnitude of the resistance phenotype when the mutations selected in WA-1 were introduced into MHV may be due to intrinsic differences between the two polymerases and the interactions between the polymerase and other replicase proteins. Although we cannot exclude a theoretical influence of the nonsynonymous mutations detected in non-replicase proteins, our current understanding of mechanism of inhibition by RDV gained from resistance selection analysis and detailed biochemical studies in several different CoVs indicates that the primary viral determinants of inhibition by RDV lie within replicase proteins, in particular the RdRp, which amino acid sequence remains highly conserved across variants of concern (VOC). Results from another in vitro SARS-CoV-2 passage study with RDV linked *nsp12*-RdRp E802D, a residue change not observed in our studies, to partial RDV resistance (*32*). GS-441524 forms an identical active metabolite to RDV in cells and acts through the same mechanism of RdRp inhibition, but may have different intracellular pharmacokinetics and triphosphate concentrations (*7*, *37*) that could theoretically contribute to differential outcomes observed in the in vitro resistance selection experiments performed with RDV versus GS-441524. Further, it will be critical to test mutations that are selected in other proteins of the CoV replicase complex, specifically in the nsp14 exonuclease, a key determinant of CoV high fidelity replication (proofreading), and to test native resistance to nucleoside analogs such as Ribavirin and 5-Fluorouracil, as well as resistance to RDV in the MHV model (*5*, *38*, *39*). Finally, it will be important to determine if the different individual and combined *nsp12*-RdRp mutations herein identified confer different extents of RDV resistance and fitness cost in SARS-CoV-2 compared to the SARS-CoV-2 lineages or in the recombinant isogenic MHV background. These direct genetic studies of SARS-CoV-2 were in process when RDV received FDA Emergency Use Authorization (EUA) and FDA Approval for treatment of COVID-19 in October 2020. The FDA approval, while stating the importance of and requiring data on SARS-CoV-2 RDV resistance determinants and potential, paradoxically triggered a halt to any newly initiated genetic studies of RDV resistance in SARS-CoV-2 using NIH or other US government support (*40*–*42*). This necessitated our targeting of reverse genetic studies using the non-human betacoronavirus MHV.

Our results also emphasize the need for additional research to determine the potential for in vivo resistance emergence and impact of resistance in various clinical settings and patient populations. A case report of an immunocompromised COVID-19 patient who responded poorly to RDV described a single mutation in *nsp12*-RdRp, but neither causal effect nor mechanism was demonstrated (*22*). Our results would predict that the barriers to RDV resistance emergence are substantial but not insurmountable. The passage-selected SARS-CoV-2 RDV-resistant lineages and the targeted engineered MHV mutants displayed either impaired replication or no advantage compared to the parallel vehicle-passaged or recombinant wild-type controls, suggesting that development of RDV resistance in SARS-CoV-2 may confer a substantial fitness cost, consistent with our previous findings on MHV RDV-resistance mutants (*5*). Further, since RDV is administered intravenously, emergence of clinical resistance during treatment of an individual likely would be disfavored by the highly controlled duration of administration and rapid and profound reduction in virus titer. Taken together, these factors would predict substantial barriers to RDV resistance in natural variants and treated patients. Our analysis of greater than 8 million consensus sequences deposited to the GISAID database also demonstrates very low prevalence in global SARS-CoV-2 isolates, including Delta and Omicron variants, of the RdRp substitutions identified in this study (*43*). Although it is encouraging that natural variants to date have not propagated confirmed RDV resistance mutations within reported consensus sequences, these substitutions might arise as minority variants. The possibility of RDV extended use in chronically infected or immunosuppressed patients also may increase the opportunities for SARS-CoV-2 to overcome genetic barriers and adapt for increased fitness.

Although our study shows that SARS-CoV-2 can develop resistance to RDV during in vitro serial passage in presence of drug and that different genetic pathways and mechanisms may be selected in parallel lineages, there are limitations to our results and interpretations. First, it is possible there may be differences in the development of drug resistance in vivo compared to in vitro, and that in vitro selected resistance may have fitness costs not defined here. Specifically, the S759A mutation was present in the clinical isolate as a low frequency variant and became dominant in two lineages under drug selection, but we do not know if that mutation would be selected if virus was passaged in vivo. Second, although we demonstrate through forward genetics in SARS-CoV-2 and reverse genetics in MHV that combined mutations S759A+V792I are sufficient to overcome sensitivity to RDV, we were restricted from reverse genetic testing of the mutations alone or together in isogenic SARS-CoV-2 making it not possible to confirm their phenotype in absence of other passage-selected mutations. Further, though we would not expect any differences in the effect of these mutations in the background of the evolving VOCs, future studies should address these questions. Finally, we did not perform detailed analysis of mutations which were selected in replicase nonstructural proteins (nsp) outside of the *nsp12*-RdRp. This important area for future genetic and biochemical studies as we seek to define additional protein functions and targets for antiviral development.

In summary, our studies present potential pathways to resistance for SARS-CoV-2 to RDV and provide multiple mechanisms of resistance that the virus uses to overcome sensitivity to RDV. Our results create a reference for surveillance for RDV resistance, and support the need to pursue combination therapies targeting the RdRp through different mechanisms (*38*, *44*), as well as inhibiting other replicase functions such as protease activities (*45*).

## MATERIALS AND METHODS

**Study Design.** The details of study design, analysis and statistics are provided in the relevant sections below. The primary goal of this study was to determine the potential pathways and mechanisms by which SARS-CoV-2 can acquire resistance to RDV. A clinical isolate of the WA1 strain of SARS-CoV-2 was serially passaged in triplicate the presence of GS-441524 or vehicle (DMSO) in Vero E6 cells to generate drug resistance. Experiments were performed in two or more independent biological replicates with two or more technical replicates each. Each virus isolate stock was given a number. Drug sensitivity assays performed in duplicate with technical replicates in A549-hACE2 cells against RDV confirmed that drug-passaged lineages showed increased resistance to RDV compared to the vehicle-passaged control populations. The researcher performing the antiviral drug sensitivity assays was blinded to the numbering at the time the experiment was performed and during data collection. Sample numbers were linked back to virus isolate names at the time of graphing and analysis to allow for data interpretation. The isolates showing phenotypic resistance at terminal passages were chosen for sequence analysis. Once the candidate mutations were identified, plaque isolation and propagation were performed to test for genetic linkages in individual plaques. Size calculations were not possible as we were working with complex virus populations and the variability of viruses within the populations could not be absolutely defined. The multiple lineages and complex passage evolution suggests that other pathways to resistance may have been possible. The goal was to best isolate mutations alone and in combination in plaque isolates. Mutations were introduced into cloned murine hepatitis virus for recovery, sequencing, and analysis of replication and RDV sensitivity in backgrounds without passage selected mutations. Candidate resistance mutations were tested by biochemical approaches using purified proteins to determine mechanisms by which these mutations result in resistance. The biochemical studies were designed to explain the mechanism of resistance of the passage-identified mutations.

**Cells and viruses.** Vero E6 cells were obtained from the United States Army Medical Research Institute of Infectious Diseases (USAMRIID) and cultured in Dulbecco’s modified Eagle medium (DMEM) (Gibco) supplemented with 10% fetal bovine serum (FBS) (Gibco), 100 U/ml penicillin (Gibco), 100 mg/ml streptomycin (Gibco), and 0.25 μM amphotericin B (Corning). A549 cells overexpressing the human ACE2 receptor (A549-hACE2) (*46*) were cultured in DMEM supplemented with 10% FBS, 100 U/ml penicillin, 100 mg/ml streptomycin, and 1% modified Eagle medium (MEM) Non-Essential Amino Acids Solution (Gibco). Murine astrocytoma delayed brain tumor (DBT) cells and baby hamster kidney 21 cells expressing the MHV receptor (BHK-R) (*47*) were maintained in DMEM containing 10% FBS (Invitrogen), 100 U/ml penicillin, 100 mg/ml streptomycin, HEPES (Gibco), and 0.25 μM amphotericin B. BHK-R cells were further supplemented with 0.8 mg/ml G418 (Mediatech). A P3 stock of the SARS-CoV-2/human/USA/WA-CDC-WA1/2020 isolate (GenBank accession no. MN985325.1) was obtained from the CDC and passaged twice in Vero E6 cells to generate a high-titer P5 stock for experiments described in this manuscript. All work with MHV was performed using the recombinant wild-type strain MHV-A59 (GenBank accession no. AY910861) (*47*). Mutant MHV viruses were generated using QuikChange mutagenesis performed according to the manufacturer’s protocol to generate mutations in MHV individual genome cDNA fragment plasmids using the previously described infectious clone reverse-genetics system (*47*). Mutants were recovered in BHK-R cells following electroporation of in vitro-transcribed genomic RNA. All fragments containing mutations were Sanger sequenced to ensure mutations were present before use in further studies (GeneWiz). RDV and GS-441524 were synthesized by the Department of Medicinal Chemistry, Gilead Sciences.

**Selection of RDV resistance.** Infection was initiated in 6-well tissue-culture plates (Corning) at a multiplicity of infection (MOI) of 0.01 plaque forming units (PFU) SARS-CoV-2 per cell in replicates of six. Three wells of Vero E6 cells were treated with 0.5 μM GS-441524, and three other wells were treated with 0.1% DMSO (vehicle controls), each well representing one lineage. Once cell monolayers demonstrated at least 40% CPE or after 72 hours, cell culture supernatant was harvested and a constant volume of 20 μL supernatant was added to a fresh monolayer to initiate the subsequent passage. All lineages were maintained until passage 13 (P13). At P13, GS-441524 lineage 2 was reduced to 3 μM to allow for virus recovery, whereas lineage 1 and 3 replicated in 9 μM GS-441524. P13 virus lineages were titered by plaque assay, and sensitivity to RDV were determined in an antiviral activity assay. In addition, RNA was harvested from infected cell supernatant using TRIzol LS reagent (Invitrogen) and cell monolayers using TRIzol reagent (Invitrogen) for viral population sequencing. Passages from GS-441524-treated lineages were subjected to viral plaque isolation by standard plaque assay in the absence of GS-441524. Plaque picks (PP) were expanded in Vero E6 cultures supplemented with 1 μM GS-441524. Cultures were harvested when CPE was greater than 50% or after 72 hours. Supernatant RNA was collected for Sanger sequencing and total monolayer RNA was harvested for RNA-seq.

**Antiviral activity assays.** A549-hACE2 cells were seeded at 5 × 10^4^ cells per well in 48-well plates (Corning) and allowed to adhere for 16 to 24 hours. RDV (20 μM in DMSO stock) was serially diluted in DMSO to achieve 1000x final concentration and diluted to final 1x concentration in culture medium up to 2 hours before start of infection. Cells were incubated with MOI = 0.01 PFU per cell of passaged virus lineages in gel saline (0.3% [wt/vol] gelatin in phosphate-buffered saline containing CaCl_2_ and MgCl_2_ (PBS +/+) (Corning) for 30 min at 37°C and gently rocked manually every 10 min to redistribute the inoculum. Viral inoculum was removed, and cells were washed with pre-warmed PBS +/+. Medium containing dilutions of RDV or vehicle control (0.1% DMSO) was added and following incubation at 37°C/5% CO_2_ for 48 hours, cell culture supernatants were harvested and processed for viral genomic RNA quantification by quantitative reverse transcription polymerase chain reaction (RT-qPCR). Data represent the means of two independent experiments consisting of two replicates each.

**Viral replication assays.** A549-hACE2 or DBT-9 cells were seeded at 1 × 10^5^ cells per well in 24-well plates (Corning) and allowed to reach confluence within 24 hours. A549-hACE2 cells were adsorbed with MOI = 0.01 PFU per ml SARS-CoV-2 passaged population virus or plaque-isolated sub-lineages. DBT-9 cells were adsorbed with MOI = 0.01 PFU per ml wild-type MHV (derived from the infectious clone) or with MHV recombinantly engineered to contain putative RDV-resistance mutations in the isogenic background. Cells were adsorbed with virus for 30 min at 37°C/5% CO_2_, with manual rocking every 10 min to redistribute the viral inoculum, after which the inoculum was removed, cells were washed with pre-warmed PBS +/+, and fresh medium without drug was added. Cultures were incubated at 37°C/5% CO_2_, supernatants were harvested at indicated times post infection, and MHV infectious titers were determined by plaque assay as previously described (*48*). Viral genomic RNA in culture supernatants was quantified by RT-qPCR.

**Quantification of SARS-CoV-2 infectious titer.** Approximately 1 × 10^6^ Vero E6 cells per well were seeded in 6-well plates and allowed to reach confluence within 24 hours. Medium was removed, and 100 μL of 10-fold serial dilutions of virus-containing supernatants in gelatin saline (0.3% [wt/vol] gelatin in PBS +/+) was adsorbed in duplicate wells for 30 min at 37°C/5% CO_2_. Plates were rocked manually every 10 min to redistribute inoculum. Cells were overlaid with DMEM containing 1% agar and incubated at 37°C/5% CO_2_. Plaques were enumerated in unstained monolayers at 48 to 72 hours post infection.

**Quantification of viral RNA.** Cell culture supernatants were harvested in TRIzol LS reagent, and RNA was purified following phase separation by chloroform as recommended by the manufacturer. RNA in the aqueous phase was collected and further purified using a KingFisher II automated nucleic acid extraction system (Thermo Fisher Scientific) according to the manufacturer’s protocol. Viral RNA was quantified by RT-qPCR on a StepOnePlus Real-Time PCR System (Applied Biosystems) using TaqMan Fast Virus 1-Step Master Mix chemistry (Applied Biosystems). SARS-CoV-2 genomic RNA was amplified and detected using forward (5′-CGTGTAGTCTTTAATGGTGTTTCC-3′) and reverse (5′-GCACATCACTACGCAACTTTAG-3′) primers and probe (5′-FAM-TTTGAAGAAGCTGCGCTGTGCAC-BHQ-1-3′) specific for the *nsp4* gene. RNA copy numbers were interpolated from a standard curve produced with serial 10-fold dilutions of *nsp4* gene RNA. Briefly, SARS-CoV-2 cloned *nsp4* gene cDNA served as template to PCR-amplify a 1062 bp product using forward (5′-TAATACGACTCACTATAGGCTGCTGAATGTACAATTTT-3′) and reverse (5′-CTGCAAAACAGCTGAGGTGATAGAG-3′) primers that appended a T7 RNA polymerase promoter to the 5′ end. PCR product was column purified (Promega) for subsequent in vitro transcription of *nsp*4 RNA using mMESSAGE mMACHINE T7 Transcription Kit (Thermo Fisher Scientific) according to the manufacturer’s protocol. *Nsp4* RNA was purified using the RNeasy mini kit (Qiagen) according to the manufacturer’s protocol, and copy number was calculated using the SciencePrimer.com copy number calculator. RNA copy numbers from MHV infections were quantified as previously described (*49*).

**Illumina sequencing.** Total RNA was extracted from P9 and P13 monolayers using TRIzol according to the manufacturer’s instructions. For RNA-Seq, total RNA underwent poly(A) selection followed by NovaSeq PE150 sequencing (Illumina) at 15 million reads per sample at the Vanderbilt University Medical Center (VUMC) core facility, Vanderbilt Technologies for Advanced Genomics (VANTAGE). Reads were aligned to the reference genome (MT020881.1), and mutations were identified, quantified, and annotated using the in-house pipeline, *CoVariant*. Amino acid locations were confirmed through sequence alignment using MacVector and CLC Workbench (QIAGEN).

**Nanopore amplicon sequencing**. 5 μL of RNA from infected cell monolayers was reverse transcribed using random hexamers and Superscript III (Thermo Fisher Scientific) to generate the first cDNA strand for each sample according to manufacturer’s protocols. *Nsp*12 amplicons 2796 bp in size were generated with first-round EasyA (Agilent) PCR using tailed primers according to manufacturer’s protocols (forward = 5′-TTTCTGTTGGTGCTGATATTGCCTGTAGATGCTGCTAAAGC-3′; reverse = 5′-ACTTGCCTGTCGCTCTATCTTCTGACATCACAACCTGGAGC-3′) and confirmed by gel electrophoresis. PCR products were purified by the Wizard SV Gel and PCR Clean-Up System (Promega) and quantified using the Qubit dsDNA HS assay (Thermo Fisher Scientific). For each sample, 1 μg of DNA (505.7 fmol) was used for barcoding PCR according to manufacturer’s protocols for the EXP-PBC001 kit (Oxford Nanopore Technologies). Barcoded amplicons were purified by the Wizard SV Gel and PCR Clean-Up System (Promega) and quantified using the Qubit dsDNA HS assay. Amplicons were pooled using 112 ng of amplicon DNA per sample for a total of 1 μg of amplicon DNA. Sequencing of the library prep was performed according to the manufacturer’s protocols using the SQK-LSK110 kit (Oxford Nanopore Technologies). The pooled library was loaded onto a quality-checked MinION flowcell with 1491 functional sequencing pores, and sequencing was performed using the *MinKNOW* GUI over 72 hours.

**Nanopore genetic linkage analysis.** Mutation linkage was determined using an in-house pipeline, *MutALink*. Analysis was directed by sequential custom Bash shell scripts that direct each module of the pipeline. The first module performs basecalling and alignment. Specifically, following sequencing, raw FAST5 files were basecalled and demultiplexed using *Guppy v5.0.11* (Oxford Nanopore Technologies). Pass FASTQ files were aligned to the SARS-CoV-2 genome (MT020881.1) for each sample using *minimap2* (*50*), and alignments were processed and filtered for reads containing sequences across all of *nsp12* using *SAMtools* (*51*). Alignment statistics were generated using *NanoStat*. The second module of the *MutALink* pipeline calls and quantifies variant allele frequencies for candidate variants using *Nanopolish* (*52*). The last module, genotype quantification, filters different combinations of candidate variants and generates outputs for each lineage using a custom batch script, variant-specific javascript files (V166A.js, N198S.js, V792I.js, S759A.js, and C799F.js), and the *samjdk* package from *jvarkit* (*53*) using a separate Bash shell script for each lineage. Read counts were corrected manually for duplicate counting between combinations, and the frequency of each genotype in each sample passage compared to total mapped reads was reported and visualized using the Python package, *seaborn* (*54*).

**Structural modeling.** The model of the SARS-CoV-2 pre-incorporation polymerase complex was built on the cryo-EM PDB structure 6XEZ (*25*) by examination of several post-incorporation/pre-translocation SARS-CoV-2 RdRp structures compared to a number of pre-incorporation complexes of similar viral RdRps (such as hepatitis C virus, norovirus, and poliovirus) (*26*–*28*). First ATP was positioned in the active site, as were two Mg^++^ ions. The corresponding template base at position +1 was modified from A to U. D618, D760 and D761 were optimized to coordinate the metal ions, and a conformational search was done on key sidechains in the active site, including K545, R553, R555, D623, S682, T687, N691, D759 and K798 (*55*). These residues, as well as metals, ATP, and primer/template nucleotides P_-1_, T_-1_ and T_+1_ were then minimized (*56*). Once optimized, ATP was modified to RDV-TP and the structure was minimized again. Mutations were analyzed by conducting a conformational search of all residues within 5 Å of the mutation and minimizing. In the case of V166A, V792I and C799R/F, a conformational search of the loops 163 to 168 and 790 to 800 was also conducted.

**Protein expression and purification**. SARS–CoV-2 RdRp wild-type and mutant proteins (S759A and V792I) were expressed and purified as reported previously (*8*, *9*, *12*). The pFastBac-1 (Invitrogen) plasmid with codon-optimized synthetic DNA sequences (GenScript) coding for a portion of 1ab polyproteins of SARS-CoV-2 (NCBI: QHD43415.1), containing only *nsp5*, *nsp7*, *nsp8*, and *nsp12*, was used as starting material for protein expression in insect cells (Sf9, Invitrogen). We employed the MultiBac (Geneva Biotech) system for protein expression in insect cells according to published protocols (*57*, *58*).

**Single NTP incorporation and the effect of primer-embedded RDV-MP**. To measure NTP incorporation by SARS-CoV-2 RdRp wild-type and mutants, data acquisition and quantification were done as previously reported (*8*, *9*, *12*). Enzyme concentration was 150 nM for both single and multiple nucleotide incorporation assays, respectively. RNA synthesis incubation time was 10 min. Single nucleotide incorporation assays were used to determine the preference for the natural nucleotide over RDV-TP. The selectivity value was calculated as a ratio of the incorporation efficiencies of the natural nucleotide over the nucleotide analog. The discrimination value was calculated as a ratio of mutant to wild-type selectivity. The efficiency of nucleotide incorporation was determined by the ratio of Michaelis–Menten constants *V*_max_ over *K*_m_ as previously reported (*8*, *9*, *12*).

**Evaluation of RNA synthesis across the RNA template with embedded RDV-MP**. RNA synthesis assays using SARS-CoV-2 RdRp complex on an RNA template with an embedded RDV-MP or adenosine at equivalent positions, data acquisition, and quantification were done as previously described with the following adjustments: (1) enzyme concentration of wild-type RdRp was increased to 250 nM, and (2) mutant RdRp concentration was adjusted such that activity was equivalent to wild-type. Two independent preparations of RDV-embedded RNA templates and at least three independent preparations of SARS-CoV-2 wild-type and mutant enzymes were used.

**Statistical analyses.** The EC_50_ value was calculated in GraphPad Prism 8 as the concentration at which there was a 50% decrease in viral replication relative to vehicle alone (0% inhibition). Dose-response curves were fit based using four-parameter non-linear regression. All statistical tests were executed using GraphPad Prism 8. Statistical details of experiments are described in the figure legends.

## Supplementary Materials

### Other Supplementary Material for this manuscript includes the following:

MDAR Reproducibility Checklist
