# Supplementary material for: Mutations in the SARS-CoV-2 RNA dependent RNA polymerase confer resistance to remdesivir by distinct mechanisms
Source: Sci Transl Med. 2022 Apr 28:eabo0718. doi: 10.1126/scitranslmed.abo0718 (PMC9097878; doi:10.1126/scitranslmed.abo0718)
Supplement: Supplementary file 1 — Figs. S1 to S3 Tables S1 and S2 [file scitranslmed.abo0718_sm.pdf]

Science Translational Medicine

Supplementary Materials for

Mutations in the SARS-CoV-2 RNA dependent RNA polymerase confer resistance to remdesivir by distinct mechanisms

Laura J. Stevens *et al.*

Corresponding author: Mark R. Denison, mark.denison@vumc.org

DOI: 10.1126/scitranslmed.abo0718

The PDF file includes:

Figs. S1 to S3
Tables S1 and S2

Other Supplementary Material for this manuscript includes the following:

MDAR Reproducibility Checklist
Data file S1

Supplementary Figures:

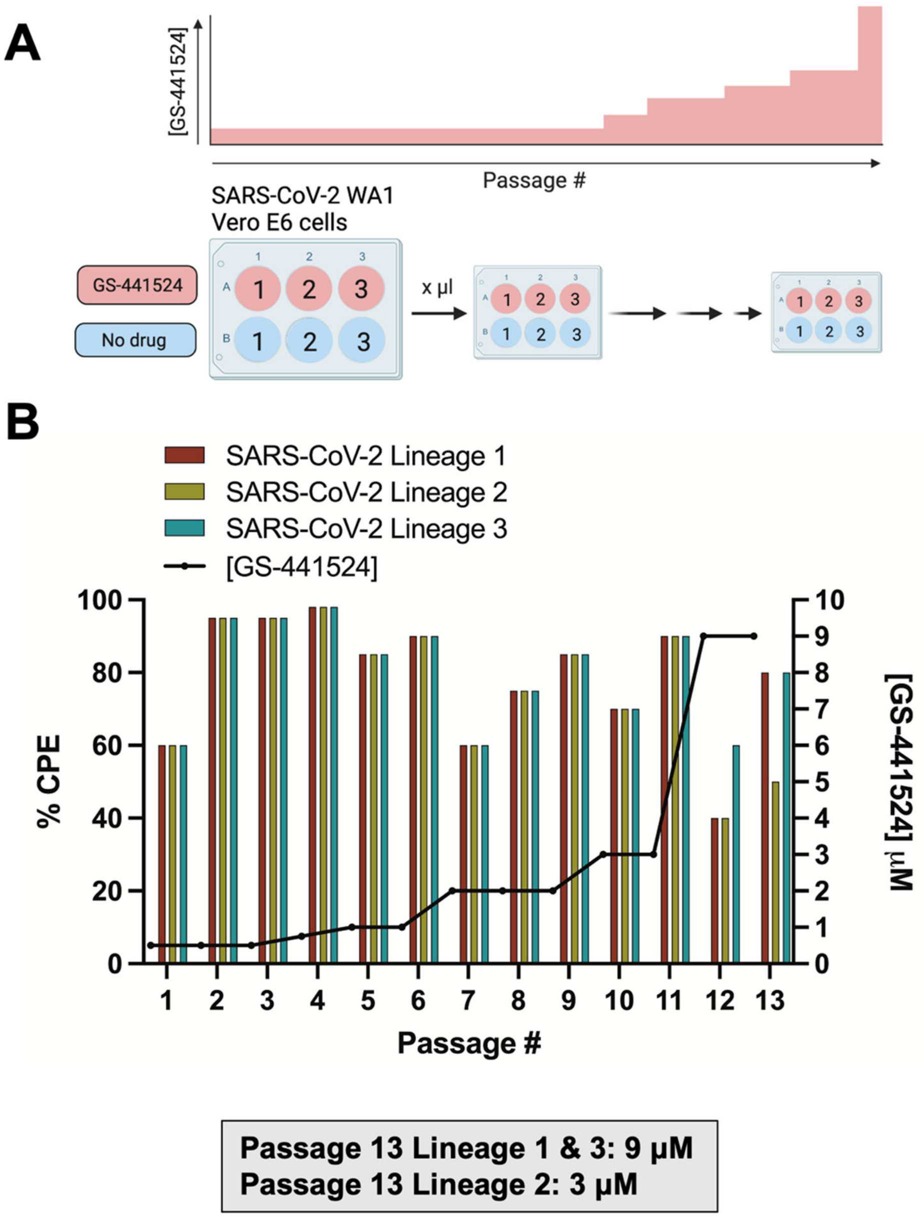

Fig. S1. Serial passaging of SARS-CoV-2 in the presence of GS-441524. SARS-CoV-2 WA-1 isolate (culture passage 5) was additionally passaged 13 times in the presence of GS-441524 or vehicle (dimethyl sulfoxide, DMSO) in Vero E6 cells (three separate lineages each). **(A)** A schematic representation of viral passaging is shown and was created with BioRender.com. **(B)** The percentage of cell monolayer with viral cytopathic effect (CPE) is shown on left Y axis and

GS-441524 concentration is shown on right Y axis and depicted with the black line. To generate passage 13 (P13), GS-441524 lineages 1 and 3 were treated with 9 μM of GS-441524, and lineage 2 was treated with 3 μM of GS-441524.

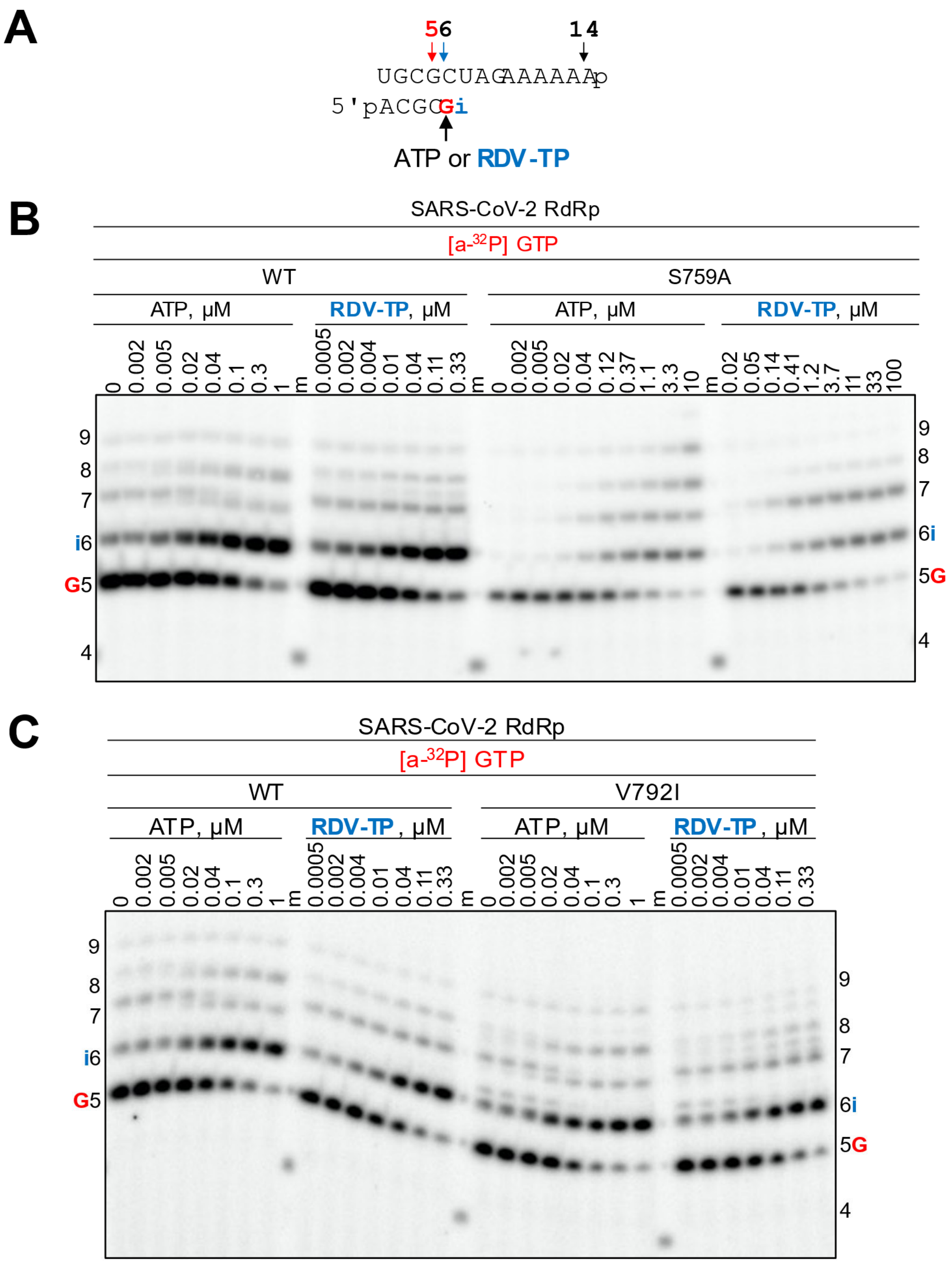

Fig. S2. Selective incorporation of remdesivir-triphosphate (RDV-TP) by wild-type and mutant (S759A and V792I) SARS-CoV-2 RNA-dependent RNA polymerase (RdRp) complexes. (A) RNA template sequence used to determine the efficiency of adenosine triphosphate (ATP) or RDV-TP incorporation at position 6 (i). G indicates incorporation of [α-^{32}P] guanosine triphosphate (GTP) at position 5 (red). **(B)** Migration patterns of the products of

ATP or RDV-TP incorporation reactions with WT and S759A SARS-CoV-2 RdRp complexes are shown. Main products emerge at position 6 and mismatches are seen at positions 7, 8, and 9. The 5'-^{32}P-labeled 4-nucleotide primer (4) is used as a size marker (m). **(C)** Migration patterns of the products of ATP or RDV-TP incorporation reactions with WT and V792I SARS-CoV-2 RdRp complexes are shown using the same conditions as described in (B).

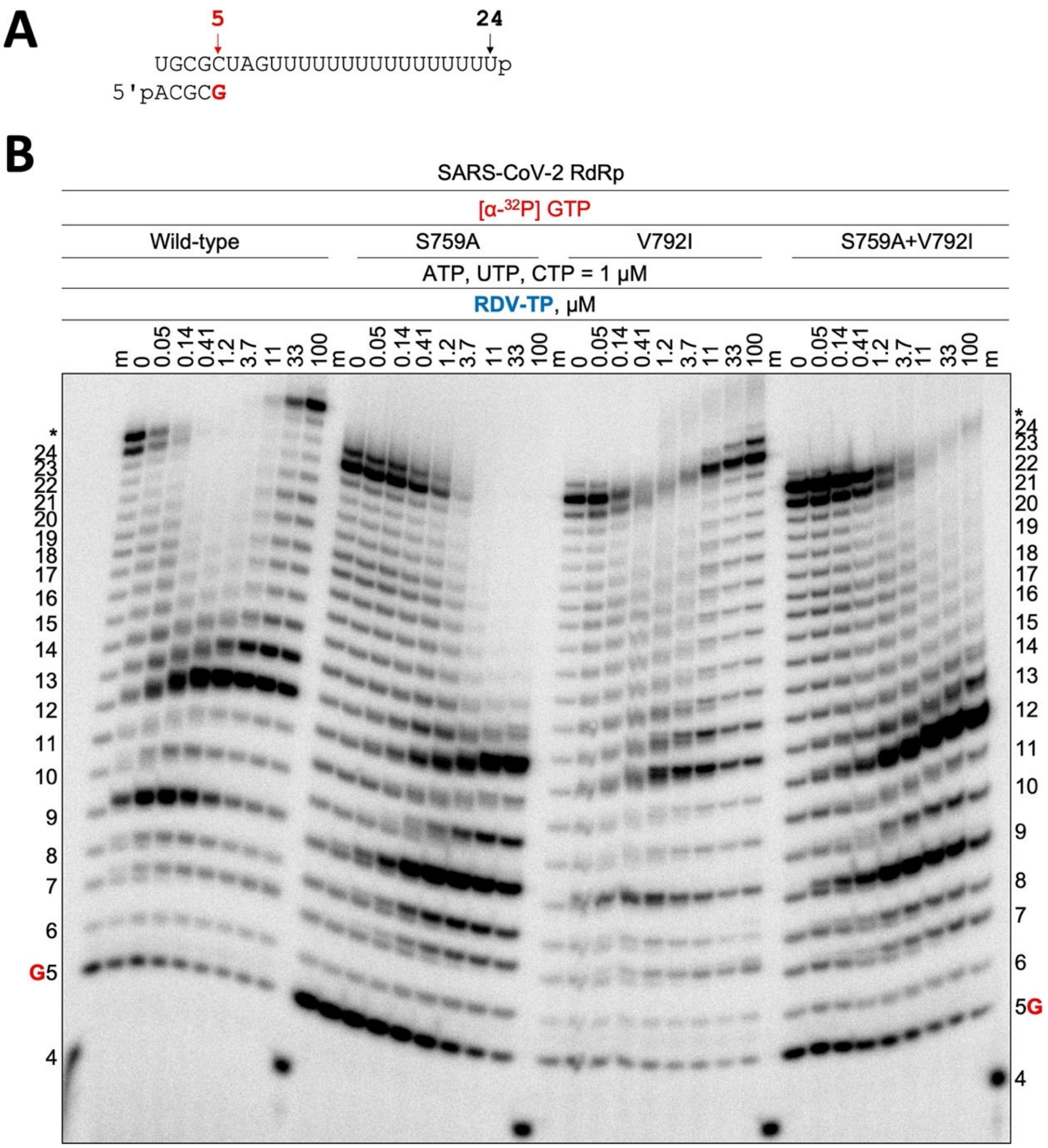

Fig. S3. Competition between RDV-TP and natural nucleoside triphosphates (NTPs) in SARS-CoV-2 wild-type and mutant S759A, V792I, and S759A/V792I RdRp complexes. (A) The RNA template sequence used is shown. **(B)** NTPs (ATP, uridine triphosphate [UTP], and cytidine triphosphate [CTP]) were supplemented at a constant concentration per reaction and RDV-TP concentrations varied as indicated. Denaturing polyacrylamide gel electrophoresis (PAGE) migration patterns of RNA products are shown. G (red) indicates the incorporation of

[α-^{32}P] GTP at position 5 and 4 indicates the migration pattern of 5'-^{32}P-labeled 4-nt primer is used as a size marker (m). The asterisk (*) indicates products formed due to terminal transferase activity.

Table S1. Half-maximal effective concentration (EC_{50}) and fold change for GS-441524 and vehicle-passaged virus lineages.

Passage	EC_{50} [mM] n=2	
**DMSO**		
P9 Lineage 1	0.36	
P9 Lineage 2	0.34	
P9 Lineage 3	0.24	
P13 Lineage 1	0.41	
P13 Lineage 2	0.39	
P13 Lineage 3	0.39	
**GS-441524**		**Fold Change**
P9 Lineage 1	0.94	2.6
P9 Lineage 2	0.56	1.5
P9 Lineage 3	0.40	1.7
P13 Lineage 1	4.22	10.4
P13 Lineage 2	1.11	2.7
P13 Lineage 3	3.27	8.0
PP nsp12-V792I	1.06	2.6
PP nsp12-S759A/V792I	2.97	7.3

SARS-CoV-2 was passaged 13 times in increasing concentrations of GS-441524 or vehicle (DMSO). Fold-change represents RDV EC_{50} ratio of drug-passaged to vehicle-passaged virus tested in A549 cells expressing human angiotensin converting enzyme 2 (A549-hACE2 cells).

Table S2. The number of times non-structural protein 12 (*nsp12*) amino acid substitutions were detected in SARS-CoV-2 sequences deposited to Global Initiative on Sharing All Influenza Data (GISAID) database.

**Residue Substitution**	**Mutation Frequency in all Sequences, % (N)** [a]	**Mutation Frequency in Omicron Sequences, % (N)** [b]	**Mutation Frequency in Delta Sequences, % (N)** [c]
V166A	0.001% (96)	0.0005% (12)	0.0003% (14)
N198S	0.01% (969)	0.003% (68)	0.016% (657)
S759A	0.000% (1)	0	0
V792I	0.002% (154)	0.003% (59)	0.0002% (9)
C799F	0.0009% (81)	0.0006% (13)	0.0006% (23)
C799R	0.000% (6)	0.00009% (2)	0.0000% (1)

[a] Total N = 8,672,594 genomes in GISAID on 03/18/22 including Omicron and Delta variants (https://www.gisaid.org).
[b] N = 2,166,224 Omicron genomes in GISAID on 03/18/22 (https://www.gisaid.org).
[c] N = 4,055,390 Delta genomes in GISAID on 03/18/22 (https://www.gisaid.org).
SARS-CoV-2 genomes with ≥ 29000nt and ≤ 5% Ns (undefined bases) were used in the analysis

Supplementary Data Files

Data file S1. Mutations present at >1% frequency in populations of serially passaged SARS-CoV-2.
